# The effect of multileaf collimator leaf width on the radiosurgery planning for spine lesion treatment in terms of the modulated techniques and target complexity

**DOI:** 10.1186/1748-717X-9-72

**Published:** 2014-03-08

**Authors:** Soo-Min Chae, Gi Woong Lee, Seok Hyun Son

**Affiliations:** 1 Department of Radiation Oncology, Cheju Halla General Hospital, Jeju, Korea; 2 Department of Radiation Oncology, Incheon St. Mary’s hospital, College of Medicine, the Catholic University of Korea, Incheon, Korea

**Keywords:** Multileaf collimator, Intensity-modulated radiotherapy, Volumetric-modulated arc therapy, Target complexity

## Abstract

**Purpose:**

We aim to evaluate the effects of multileaf collimator (MLC) leaf width (5 mm vs. 2.5 mm) on the radiosurgery planning for the treatment of spine lesions according to the modulated techniques (intensity-modulated radiotherapy [IMRT] vs. volumetric-modulated arc therapy [VMAT]) and the complexity of the target shape.

**Methods:**

For this study, artificial spinal lesions were contoured and used for treatment plans. Three spinal levels (C5, T5, and L2 spines) were selected, and four types of target shapes reflecting the complexity of lesions were contoured. The treatment plans were performed using 2.5-mm and 5-mm MLCs, and also using both static IMRT and VMAT. In total, 48 treatment plans were established. The efficacy of each treatment plan was compared using target volume coverage (TVC), conformity index (CI), dose gradient index (GI), and V_30%_.

**Results:**

When the 5-mm MLC was replaced by the 2.5-mm MLC, TVC and GI improved significantly by 5.68% and 6.25%, respectively, while CI did not improve. With a smaller MLC leaf width, the improvement ratios of the TVC were larger in IMRT than VMAT (8.38% vs. 2.97%). In addition, the TVC was improved by 14.42-16.74% in target type 4 compared to the other target types. These improvements were larger in IMRT than in VMAT (27.99% vs. 6.34%). The V_30%_ was not statistically different between IMRT and VMAT according to the MLC leaf widths and the types of target.

**Conclusion:**

The smaller MLC leaf width provided improved target coverage in both IMRT and VMAT, and its improvement was larger in IMRT than in VMAT. In addition, the smaller MLC leaf width was more effective for complex-shaped targets.

## Background

Spine metastasis can cause intractable pain, and when it damages the spinal cord, it can lead to motor and/or sensory dysfunction. Consequently, spine metastasis is the primary cause of deterioration in the quality of life of cancer patients [1,2]. The treatment of spine metastasis includes surgery, systemic chemotherapy, radiotherapy, as well as medical treatment with analgesics and steroids; most patients undergo radiotherapy. In the past, radiotherapy consisted of delivering modest doses of radiation to spinal lesions using conventional techniques, but through advances in planning and delivery techniques, radiosurgery, which can deliver highly localized doses of radiation, is now being widely used [2-4]. Because spinal lesions are only slightly affected by respiration or internal organ movement, those are suitable for radiosurgery, which is effective in reducing pain [3,5-8].

Three-dimensional conformal radiotherapy (3D-CRT), dynamic conformal arc therapy (DCAT), and intensity-modulated radiotherapy (IMRT) are commonly used in radiosurgery. Since the recent development of volumetric-modulated arc therapy (VMAT), in which the IMRT technique is combined with the DCAT technique, several studies have been conducted on it [3,5,9-12]. The multileaf collimator (MLC), which is used for linac-based radiosurgery, continues to be developed, and is now commercially available with a 2.5-mm leaf width. Several studies using 3D-CRT, DCAT, and IMRT have reported that smaller MLC leaf width provides dosimetric improvement, particularly in the radiosurgery for small lesions, and the use of micro-MLC, which has a leaf width of less than 5 mm, has provided good results [13-16]. The differences in VMAT planning related to different MLC leaf width sizes (5 mm vs. 2.5 mm) have not been evaluated. Furthermore, the effects of a smaller MLC width have not been evaluated by comparing VMAT with other techniques. The existing researches investigated the effects of MLC leaf width size in relation to the target shape [16-18]. However, these researches were limited because the target complexities were not methodically classified.

In this study, we evaluated the difference in dosimetric effects between 5-mm and 2.5-mm MLC leaf width in radiosurgery of spine lesions. We established the change in the quality of the dose distribution for the modulated techniques (VMAT and IMRT) and verified the effects of MLC leaf width in relation to the complexity of the target shape.

## Methods

### Target delineation

This study was designed to evaluate the dosimetric effects of different MLC leaf widths on the quality of the dose distribution in relation to the radiotherapy techniques and target shape complexity. Therefore, artificial spine lesions were contoured and used for treatment plans.

For the simulations, patients were immobilized using a thermoplastic head mask for C spine and the BodyFix system (Medical Intelligence, GmbH, Schwabmuenchen, Germany) for T and L spines. Spiral computed tomography (CT) scans were performed using the Ingenuity 128-channel CT scanner (Philips Healthcare, Eindhoven, The Netherlands) with a 1-mm slice thickness. The patients’ CT data were retrospectively reviewed and used for this planning study following institutional review board approval (IRB of Incheon St. Mary’s Hospital, the Catholic University of Korea, Reference number: OC13RISI0061). Written informed consent was obtained from the patient for the publication of this report and any accompanying images.

**Figure 1:**
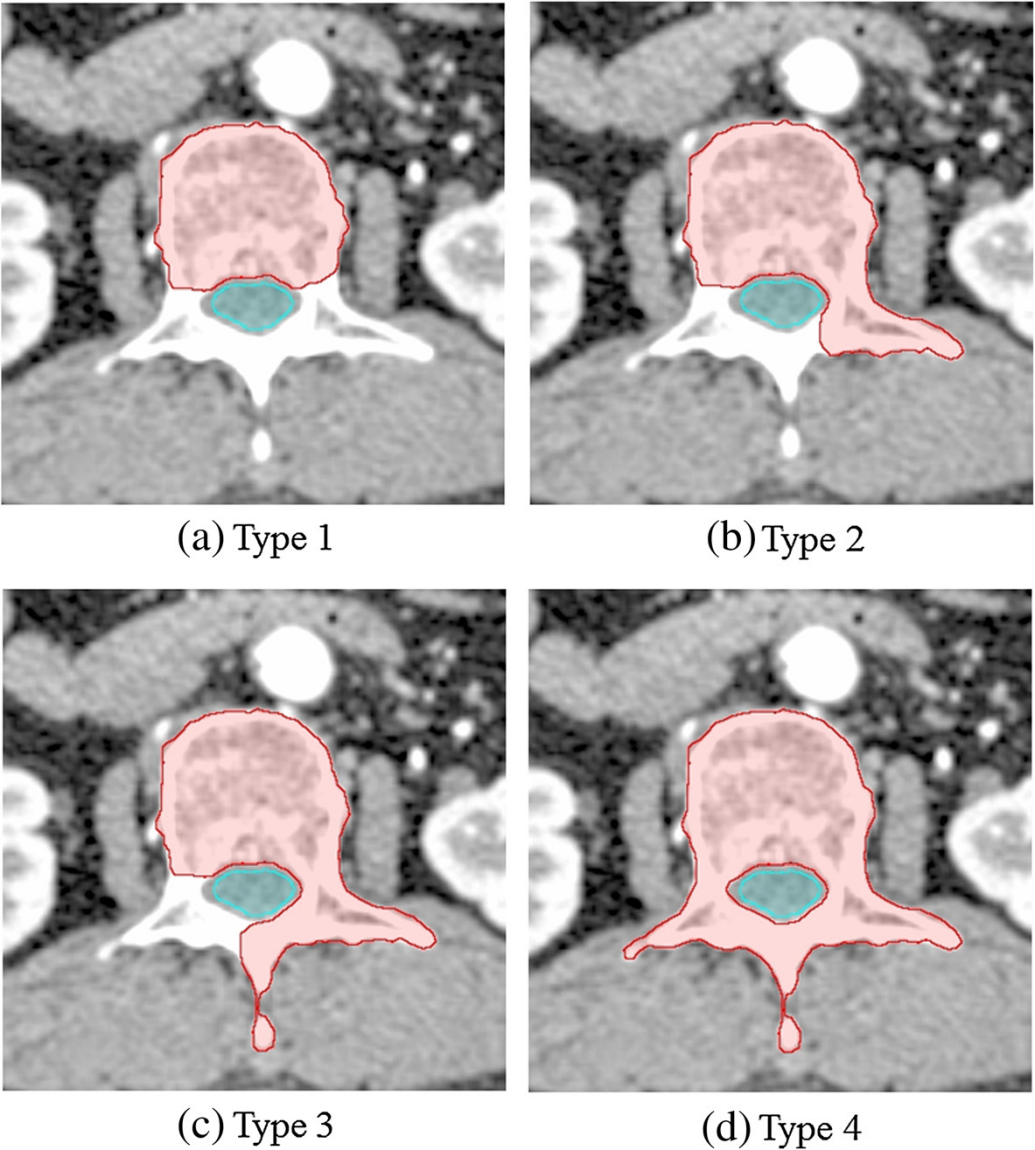
**Four types of target shapes, reflecting the complexity of lesions. (a)** Type 1, **(b)** Type 2, **(c)** Type 3, and **(d)** Type 4.

To represent various shapes of spinal lesions, three spinal levels (C5, T5, and L2) were selected, and four types of target shapes, reflecting the complexity of the lesions, were contoured (Figure 1). The four types of spinal lesions were contoured by modifying a Weinstein, Boriani, and Biagnini (WBB) surgical staging system, which provides various degrees of spinal lesions surrounding the spinal cord [19-21]. Type 1 is defined as the entire vertebral body only (WBB zones 4, 5, 6, 7, 8, and 9), and type 2 is defined as the vertebral body and left transverse process (WBB zones 4, 5, 6, 7, 8, 9, 10, and 11). Type 3 is defined as the vertebral body, left transverse process, and spinous process (WBB zones 1, 4, 5, 6, 7, 8, 9, 10, 11, and 12), and type 4 is defined as the vertebral body, left transverse process, spinous process, and right transverse process (entire spinal cord encompassed state, WBB zones 1–12). The spinal cord is defined as the spinal canal minus 1 mm from the entire circumference, as the gap between the target and the spinal cord was 1 mm. The spinal cord was contoured by extending 10 mm both above and below the level of the target volume. Relevant structures such as the esophagus, lungs, and both kidneys were also contoured. The target volumes and spinal cord volume in accordance with the level of the spine and the types of target shapes are summarized in Table 1.

**Table 1:** Target and spinal cord volumes

	**Target (cc)**				**Spinal cord (cc)**
	**Type 1**	**Type 2**	**Type 3**	**Type 4**	
C5	4.62	6.37	7.67	9.90	7.37
T5	14.19	18.60	23.18	27.69	9.14
L2	30.32	35.02	38.98	44.45	9.76

### Prescription and radiation treatment planning

The prescribed dose was 18 Gy in a single fraction, with a maximum dose of 10 Gy allowed to 0.25 cc of spinal cord [22]. In this study, we intended to obtain the maximal target volume coverage satisfying the dose constraint for the spinal cord. Therefore, the irradiated dose to 0.25 cc of spinal cord is 10 Gy, which is the same for all types of plans. However, the coverage rates of the prescribed dose to the target volume varied according to each plan.

All treatment plans were performed with Eclipse version 8.9 (Varian Medical Systems, Palo Alto, CA) to exclude any bias due to the effect of different planning algorithms. The Dose Volume Optimizer version 8.9 for IMRT and the Progressive Resolution Optimizer version 8.9 for VMAT were used for the plan optimization. The Anisotropic Analytic Algorithm version 8.9 was used for the dose calculations. The treatment plans were performed using a 2.5-mm and 5-mm MLC and using both multiple static field sliding window IMRT and multi-arc VMAT. For each plan, the same parameters such as the isocenter location; the number of fields; the MLC margin; the gantry, collimator, and couch angles for each beam; and the dose constraints level were used. The IMRT plans consisted of single-isocenter, coplanar, and 11 fields delivered by the sliding-window method (dynamic MLC mode). The fluence map pixel size was 2 × 2 mm^2^. The whole angles were 0°, 33°, 65°, 98°, 130°, 163°, 195°, 228°, 260°, 293°, and 326°. The VMAT plans were implemented with a single-isocenter 3-full arc, and without a couch rotation. The collimator angles were set to 45°, 315°, and 90° for each arc. The gantry angle range of each arc was 179.9°-181.1°. Several studies have reported that static IMRT planning using more than 7 fields results in a dosimetric gain [5,23], and that VMAT planning using multi-arc, rather than 1-arc, achieves better results [3,24]. Therefore, in this study, for the best results for the two different planning techniques, we use an 11-field static IMRT and a 3-arc VMAT.

**Figure 2:**
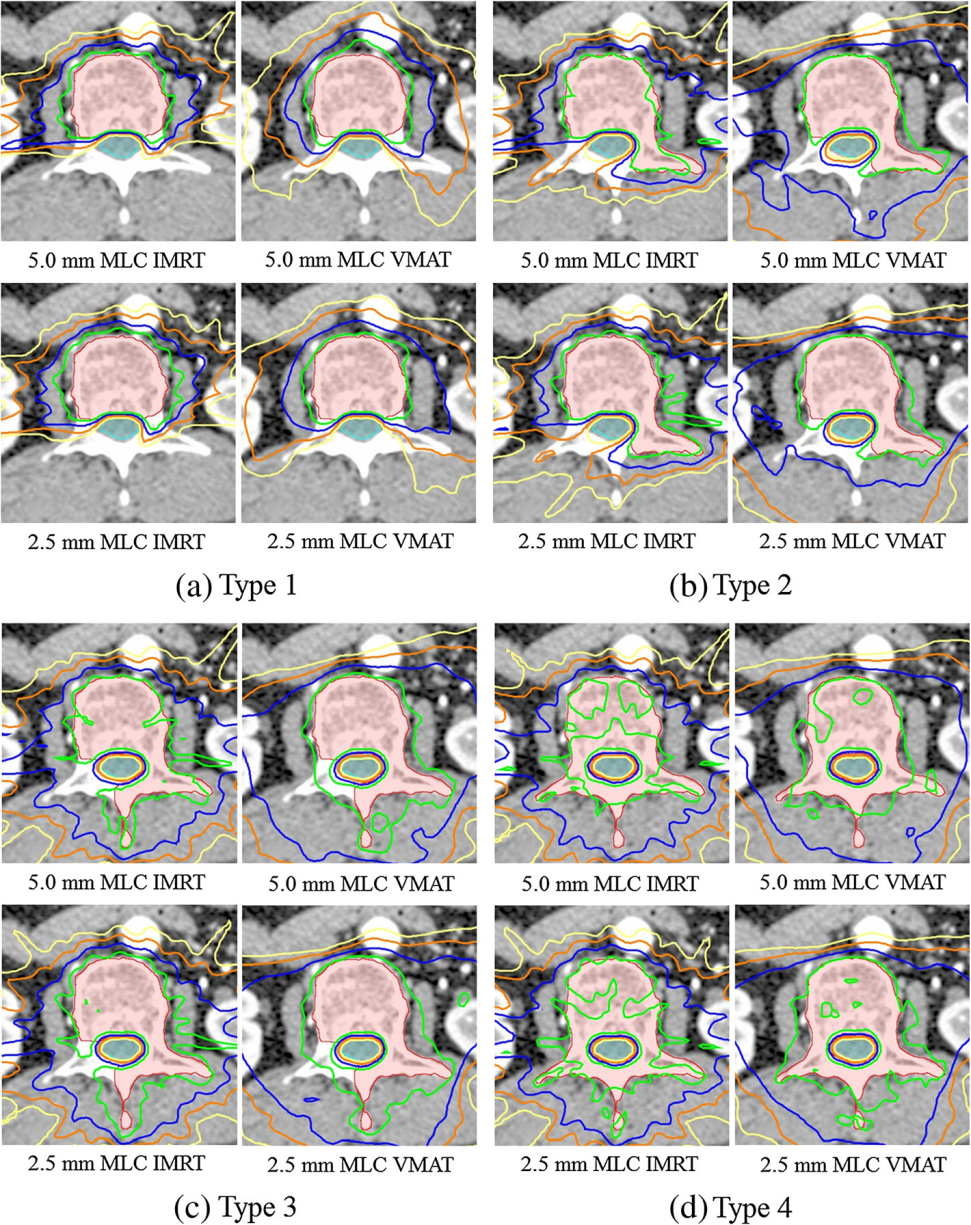
**Established treatment plans according to the four types of target shapes, two MLC leaf widths, and two types of planning techniques. (a)** Type 1, **(b)** Type 2, **(c)** Type 3, and **(d)** Type 4 (the green line is the 18 Gy isodose line, blue line is the 15 Gy isodose line, orange line is the 12 Gy isodose line, yellow line is the 10 Gy isodose line).

Thus, 48 treatment plans were established according to the three types of spine levels, four types of target shapes, two MLC leaf widths, and two types of planning techniques. The established treatment plans are shown in Figure 2.

### Dosimetric indices

Target volume coverage (TVC), conformity index (CI), dose gradient index (GI), and V_30%_ were used to compare the efficacy of each treatment plan. The definition of each index is summarized below.

1. TVC: The index to evaluate the dose coverage of the target volume [25,26]. A larger value of TVC indicates a better dose coverage of the target volume.

$$\begin{array}{c} \text{Volume within the target receiving}\\ \mathrm{TVC}\left(\%\right)=\frac{\text{at least the prescription isodose}}{\mathrm{Target}\;\mathrm{volume}}\times 100\left(\%\right) \end{array}$$

2. CI: The ratio used to evaluate the quality of fit of the target volume to the prescription isodose volume. It was proposed by the Radiation Therapy Oncology Group (RTOG) and modified by Paddick et al. [27] and Nakamura et al. [28]. A smaller value of CI indicates a better conformity of the target volume.

$$\mathrm{Conformity}\;\mathrm{index}\left(\mathrm{CI}\right)=\frac{\mathrm{PIV}\times \mathrm{TV}}{{\mathrm{PTV}}_{\mathrm{PIS}}\times {\mathrm{PTV}}_{\mathrm{PIS}}}$$

[PIV, prescription isodose volume; PTV_PIS_, planning target volume encompassed within the prescription isodose surface; TV, target volume]

3. GI: The index that represent the degree of dose drop-off outside the target volume, which was proposed by Paddick et al. [29]. A smaller value of GI indicates a better degree of dose drop-off outside the target volume.

$$\mathrm{Dose}\;\mathrm{gradient}\;\mathrm{index}\left(\mathrm{GI}\right)=\frac{V_{50}}{\mathrm{PT}V_{\mathrm{PIS}}}\times 100\left(\%\right)$$

[V_50_, volume receiving at least 50% dose of the prescription dose; PTV_PIS,_ planning target volume encompassed within the prescription isodose surface]

4. Improvement ratio: The ratio used to evaluate the improvement in the index between the two rival plans (a plan with a 2.5-mm MLC vs. a plan with a 5-mm MLC) [30].

$$\begin{array}{l} \mathrm{Improvement}\;\mathrm{ratio}\left(\%\right)=\frac{\left(\mathrm{Inde}x_{2.5-\mathrm{mm}\;\mathrm{MLC}}-\mathrm{Inde}x_{5-\mathrm{mm}\;\mathrm{MLC}}\right)}{\mathrm{Inde}x_{5-\mathrm{mm}\;\mathrm{MLC}}}\\ \;\times 100\left(\%\right) \end{array}$$

5. V_30%_: The irradiated volume receiving more than 30% of the prescription dose.

### Statistical analysis

An independent *t*-test was used to analyze the influence of the MLC size, and the difference according to the types of target shapes was evaluated using a one-way analysis of variance test. A Wilcoxon signed rank test was used to compare the low dose distribution (V_30%_) between IMRT and VMAT plans according to the MLC leaf width and the types of target. The statistical analysis was conducted using MedCalc version 12.6 for Windows (MedCalc Software, Ostend, Belgium) and a *p* value <0.05 was considered statistically significant.

## Results

### Comparison between 2.5-mm and 5-mm MLCs in IMRT and VMAT plans

The dosimetric indices and their improvement ratios according to the MLC leaf width (2.5 mm vs. 5 mm) and modulated techniques (IMRT vs. VMAT) are summarized in Table 2. The D_0.25cc_ of the spinal cord was a median of 10.00 Gy (range: 9.97-10.02 Gy), which was not statistically different in relation to the MLC type, planning technique (IMRT vs. VMAT), types of target shapes, and spine level (*p* = 0.087, 0.087, 0.994, and 0.471, respectively).

**Table 2:** Dosimetric indices and their improvement ratios according to the MLC leaf width (2.5 mm vs. 5 mm) and intensity-modulated techniques (IMRT vs. VMAT)

		**2.5-mm MLC**	**5-mm MLC**	**Improvement ratio (%)**	***p *** **value**
Overall	TVC	91.83 ± 11.56	88.10 ± 15.23	5.68 ± 10.07	0.003
	CI	1.94 ± 0.53	2.06 ± 0.81	−2.42 ± 12.25	0.110
	GI	9.99 ± 2.13	10.89 ± 2.81	−6.25 ± 18.84	0.023
IMRT	TVC	88.40 ± 15.62	83.55 ± 20.24	8.38 ± 13.66	0.042
	CI	2.03 ± 0.67	2.24 ± 1.06	−4.86 ± 13.00	0.119
	GI	9.30 ± 2.06	10.98 ± 3.34	−13.79 ± 7.38	0.003
VMAT	TVC	95.26 ± 3.12	92.65 ± 5.48	2.97 ± 3.10	0.005
	CI	1.85 ± 0.34	1.88 ± 0.41	0.02 ± 11.48	0.689
	GI	10.68 ± 2.04	10.80 ± 2.30	1.27 ± 23.74	0.871

**Figure 3:**
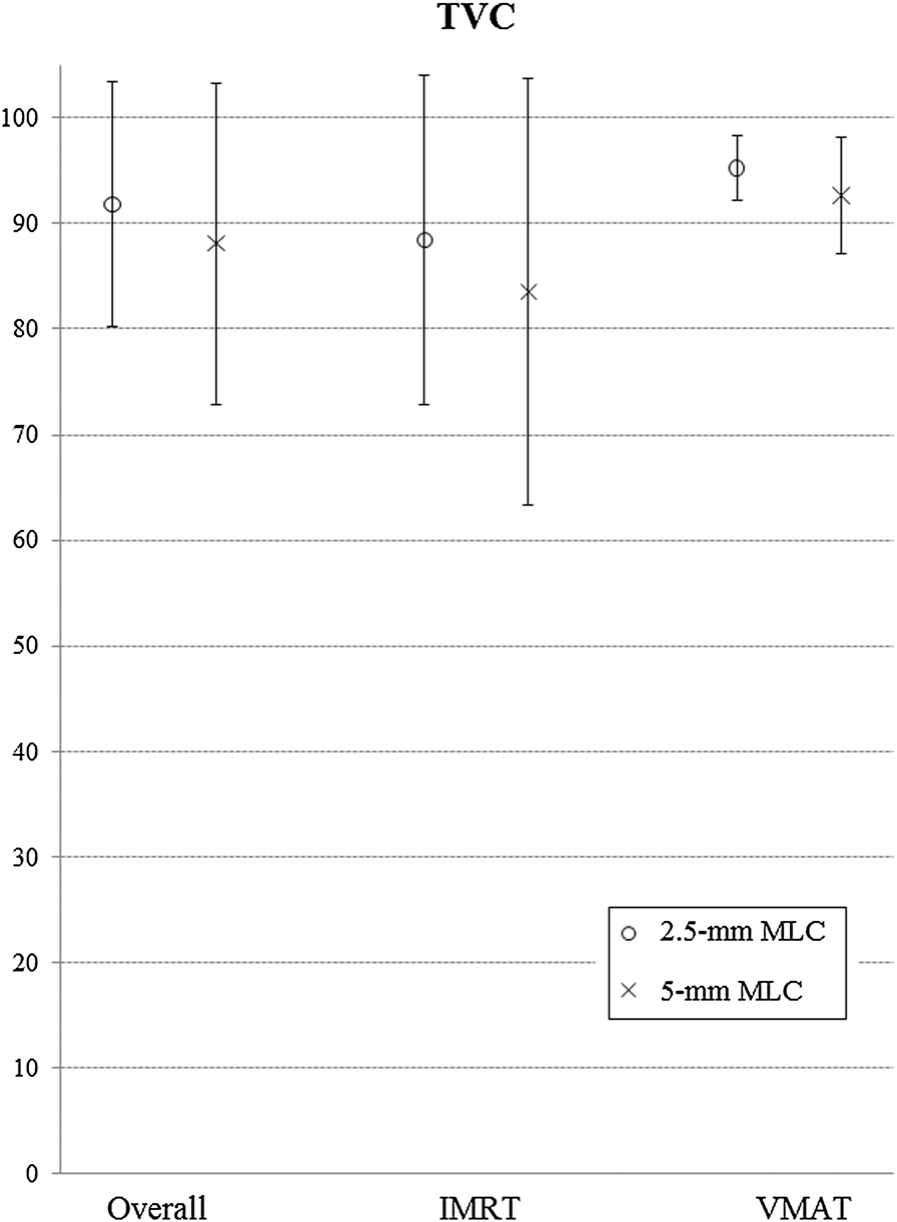
Comparison of TVC between 2.5-mm and 5-mm MLC in IMRT and VMAT plans.

The mean TVC was 88.10% with the 5-mm MLC and 91.83% with the 2.5-mm MLC. When using the 2.5-mm MLC instead of the 5-mm MLC, TVC was improved by 5.68%, which was statistically significant (*p* = 0.003). In IMRT, the mean TVC was 83.55% with the 5-mm MLC and 88.40% with the 2.5-mm MLC, and the improvement ratio was 8.38%. In VMAT, the mean TVC was 92.65% with the 5-mm MLC and 95.26% with the 2.5-mm MLC, and the improvement ratio was 2.97%. TVC was higher in VMAT than in IMRT, and the improvement ratio was higher in IMRT (Figure 3).

The mean CI was 2.06 with the 5-mm MLC and 1.94 with the 2.5-mm MLC; this difference is not statistically significant (*p* = 0.110). In the case of IMRT, the mean CI was 2.24 with the 5-mm and 2.24 with the 2.5-mm MLC. In the case of VMAT, the mean CI was 1.88 with the 5-mm and 1.85 with the 2.5-mm MLC. There is no statistically significant difference between the MLC leaf widths (*p* = 0.042 and 0.689, respectively). CI was larger in VMAT than in IMRT.

The mean GI was 10.89 with the 5-mm MLC and 9.99 with the 2.5-mm MLC. Comparing the 2.5-mm with the 5-mm MLC, GI was improved by 6.25%, which is statistically significant (*p* = 0.023). In IMRT, the mean CI was 10.98 and 9.30 with the 5-mm and the 2.5-mm MLC, respectively, and the improvement ratio was 13.79%, which is statistically significant (*p* = 0.003). In VMAT, the mean GI was 10.80 and 10.68 with the 5-mm and the 2.5-mm MLC, respectively, and the difference is not statistically significant (*p* = 0.871).

### Comparison of V_30%_ between IMRT and VMAT plans according to the MLC leaf widths and the types of target

To evaluate the low dose distribution of the IMRT and VMAT plans, the V_30%_ values are compared according to the MLC leaf widths and the types of target (Table 3). The mean V_30%_ was 460.80 cm^3^ and 466.44 cm^3^ in the IMRT and VMAT plans, respectively; the difference is not statistically significant (*p* = 1.000).

**Table 3:** **Comparison of V**_**30% **_**between IMRT and VMAT plans according to the MLC leaf width and the types of target**

		**IMRT (cm**^**3**^**)**	**VMAT (cm**^**3**^**)**	***p *** **value**
MLC	2.5-mm MLC	455.84 ± 316.45	466.10 ± 288.69	0.875
	5.0-mm MLC	465.76 ± 315.02	466.78 ± 276.48	0.875
Target shapes	Type 1	315.33 ± 216.79	380.36 ± 257.99	0.075
	Type 2	439.84 ± 287.24	470.90 ± 290.22	0.173
	Type 3	551.47 ± 368.23	514.65 ± 305.96	0.249
	Type 4	536.55 ± 361.91	499.86 ± 296.27	0.249
Overall		460.80 ± 308.84	466.44 ± 273.95	1.000

In the 2.5-mm MLC, the mean V_30%_ was 455.84 cm^3^ and 466.10 cm^3^ in the IMRT and VMAT plans, respectively. In the 5.0-mm MLC, the mean V_30%_ was 465.76 cm^3^ and 466.78 cm^3^ in the IMRT and VMAT plans, respectively. In both types of MLC, there was no statistically significant difference (*p* = 0.875 for 2.5-mm and 0.875 for 5.0-mm MLC).

According to the types of target, the mean V_30%_ for IMRT and VMAT were, respectively, 315.33 cm^3^ and 380.36 cm^3^ for type 1, 439.84 cm^3^ and 470.90 cm^3^ for type 2, 551.47 cm^3^ and 514.65 cm^3^ for type 3, and 536.55 cm^3^ and 499.86 cm^3^ for type 4. For all target types, there was no statistically significant difference (*p* = 0.075, 0.173, 0.249, and 0.249, respectively).

### Improvement ratio according to the types of target

The improvement ratios (the 2.5-mm MLC compared to the 5-mm MLC) of the dosimetric indices according to the types of target shapes in IMRT and VMAT are summarized in Table 4.

**Table 4:** Improvement ratios of the dosimetric indices according to the types of target in IMRT and VMAT

		**Improvement ratio (%)**				
		**Type 1**	**Type 2**	**Type 3**	**Type 4**	***p *** **value**
Overall	TVC	0.42 ± 1.01	2.39 ± 5.24	2.74 ± 2.80	17.16 ± 14.68	0.006
	CI	−0.41 ± 9.34	−5.58 ± 6.61	3.77 ± 9.00	−7.46 ± 19.63	0.396
	GI	8.89 ± 33.04	−10.61 ± 4.40	−7.85 ± 5.31	−15.46 ± 9.52	0.122
IMRT	TVC	−0.16 ± 0.36	3.62 ± 7.97	2.10 ± 2.32	27.99 ± 13.35	0.007
	CI	−4.97 ± 8.94	−6.83 ± 4.75	7.16 ± 9.91	−14.81 ± 18.92	0.231
	GI	−8.85 ± 4.59	−14.30 ± 2.43	−9.37 ± 5.68	−22.62 ± 7.57	0.047
VMAT	TVC	1.01 ± 1.18	1.15 ± 0.78	3.37 ± 3.61	6.34 ± 3.11	0.094
	CI	4.14 ± 8.72	−4.33 ± 9.06	−0.39 ± 8.35	−0.11 ± 21.06	0.883
	GI	26.64 ± 41.98	−6.92 ± 1.32	−6.33 ± 5.59	−8.29 ± 3.92	0.208

**Figure 4:**
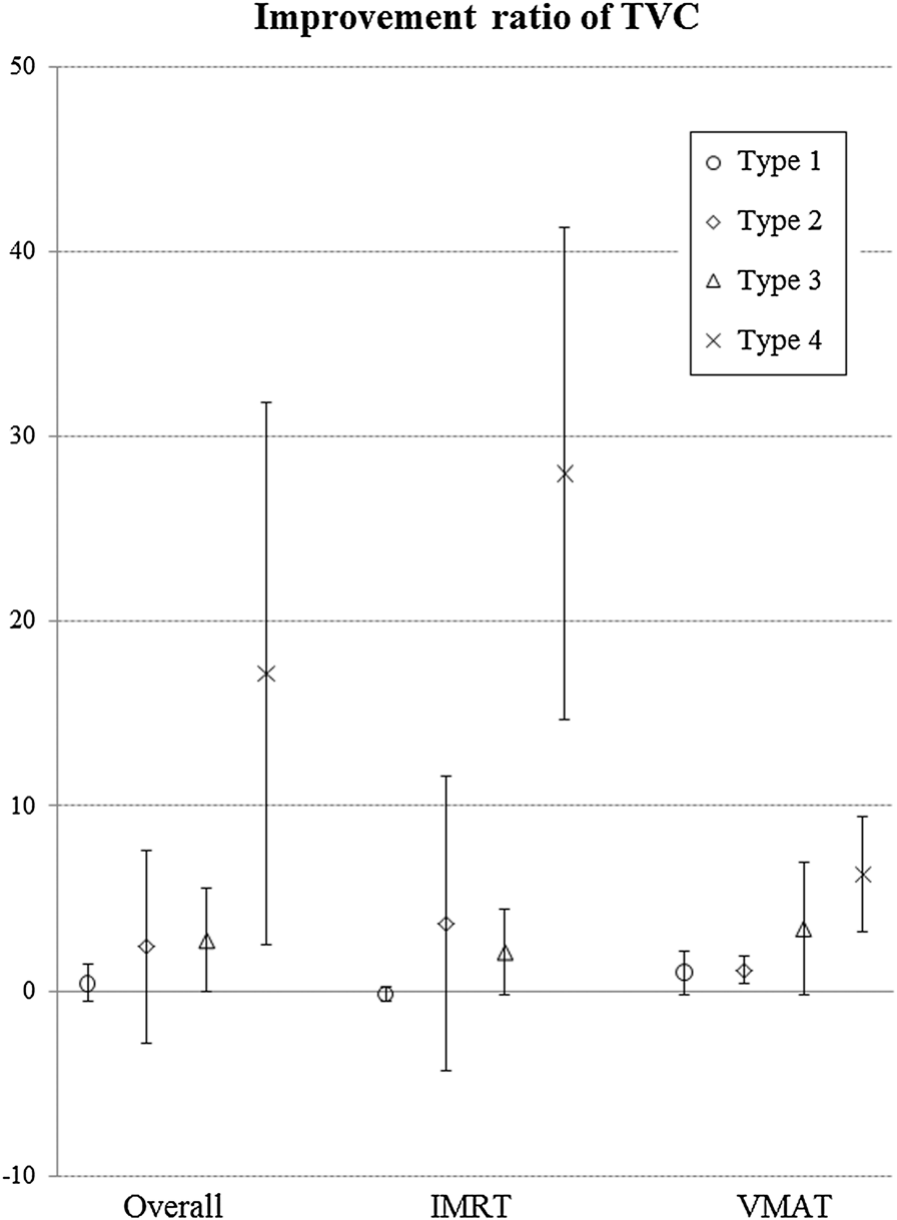
Improvement ratio of TVC according to the types of target.

The mean improvement ratio of TVC was 0.42% in type 1, 2.39% in type 2, 2.74% in type 3, and 17.16% in type 4. There was a statistically significant difference in type 4 compared with other types (1, 2, and 3) (*p* = 0.006), and the difference in the improvement ratio was 14.42-16.74%. In IMRT, the improvement ratios of Type 1, 2, 3, and 4 were −0.16%, 3.62%, 2.10%, and 27.99%, respectively. In VMAT, the improvement ratios for types 1, 2, 3, and 4 were 1.01%, 1.15%, 3.37%, and 6.34%, respectively (Figure 4). A statistical difference was observed only in IMRT (*p* = 0.007).

The mean improvement ratio of CI was −0.41% in type 1, −5.58% in type 2, 3.77% in type 3, and −7.76% in type 4. However, there was no statistically significant difference between different target shapes (*p* = 0.396), and there was no statistically significant difference between IMRT and VMAT (*p* = 0.231 and 0.883, respectively).

The mean improvement ratio of GI was 8.89% in type 1, −10.61% in type 2, −7.85% in type 3, and −15.46% in type 4. However, there was no statistically significant difference (*p* = 0.122). In IMRT, the mean improvement ratios of types 1, 2, 3, and 4 were −8.85%, −14.30%, −9.37%, and −22.62%, respectively, and a statistically significant difference was observed (*p* = 0.047). However, there was no statistically significant difference in VMAT (*p* = 0.208).

## Discussion

Owing to developments in MLCs in addition to improvements in radiotherapy planning techniques and delivery methods, radiosurgery has been widely used, and many studies have reported its dosimetric superiority and clinical effectiveness [3,5-8]. Jin et al. reported that when a dose of 18 Gy was prescribed, and 10% of the adjacent spinal cord volume was irradiated below 10 Gy, 85% of patients safely experienced pain relief and neurological improvement [4].

The micro-MLC commonly used for radiosurgery is defined as having a leaf width of less than 5 mm. Galal et al. tested the dosimetric and mechanical characteristics of a 3-mm micro-MLC and concluded that it was suitable for use in radiosurgery [31]. Many investigators have studied the effects of MLC leaf width in various planning techniques (3D-CRT and IMRT). Monk et al. compared 3-mm and 5-mm MLC in radiosurgery with the 3D-CRT technique for 14 brain lesions [13]. CI was better with the 3-mm MLC, but there was no statistically significant improvement in the index for organs at risk (D_max_ to the critical structures). Kubo et al. found, while studying the difference of MLC size in radiosurgery with 3D-CRT for prostate cancer, that when a 10-mm MLC was replaced with a 3-mm MLC, the dose to the bladder and rectum was lowered, but CI was not improved [17]. Wu et al. compared IMRT plans using 5-mm and 2.5-mm MLCs for 15 cases of brain, liver, and spine lesions. In the brain lesion, the indices for target volume such as D_min_, D_max_, and the homogeneity index (HI) were improved, and in the spine lesion, the indices for organs at risk such as D_1_, D_10_, and the mean dose to the cord were improved by 14%, 19%, and 29%, respectively [16]. Wang et al. compared IMRT plans using 10-mm and 4-mm MLCs for 10 patients with prostate cancer, and both the indices for target volume (D_max-1cc_, D_99_, and HI), and dose to the rectum and bladder were improved significantly [32,33]. According to Jin et al. and Dvorack et al., CI and TVC were improved significantly when the MLC leaf width was changed from 10 mm to 3 mm in the IMRT plans [14,34]. However, according to Burmeister et al., when they compared a 10-mm MLC with a 5-mm MLC in the IMRT plans, there were no significant differences in D_min_, D_max_, and D_mean_. The treatment efficacy was lower for the 5-mm MLC because of an increase in the whole body dose, the treatment time, and the monitor unit [35].

These effects of the MLC leaf width varied according to the radiotherapy technique used. The benefits of a small MLC were more prominent in the 3D-CRT compared to the IMRT technique [14,34,36]. Tanyi et al. analyzed the impact of the MLC leaf width difference (2.5 mm vs. 5 mm) on the 3D-CRT, DCAT, and IMRT techniques for 68 cases of brain lesions. In DCAT, the indices for target volume (CI, D_min_, D_max_, and D_mean_) and index for organs at risk (PRV100, peritumoral rind volume receiving ≥100% of the prescription dose) were improved, while in 3D-CRT, only the indices for target volume (CI and D_min_) were improved. However, in IMRT, there was no improvement in the indices for target volume (CI, D_min_, D_max_, and D_mean_) and organs at risk (PRV100) [36].

Recently, the VMAT technique was developed by combining IMRT with the concept of dynamic arc therapy, and many authors have reported results comparing static IMRT and VMAT [2,3,24,37]. The studies of 7-field IMRT compared with VMAT showed that 1-arc VMAT was similar to or slightly more effective than IMRT [2,37]. Lee et al. compared 7-field IMRT and 1-arc VMAT plans for 5 cases of spine lesions and reported that 1-arc VMAT was better than 7-field IMRT in CI and D_90_, and the dose to the spinal cord was significantly lower in 1-arc VMAT (*p* = 0.04) [2]. Tsai et al. compared 7-field IMRT with 1-arc VMAT for 12 patients with prostate cancer, and 1-arc VMAT showed better result than 7-field IMRT both in Nakamura’s CI and dose to the rectum [37]. Reports have shown 2-arc VMAT to be superior to IMRT, but if the number of fields is increased in IMRT, it has been reported to be better than 1-arc VMAT and similar to 2-arc VMAT [3,24]. Roa et al. compared 7–14 fields IMRT, 1-arc VMAT, and 2-arc VMAT plan for 23 cases of brain and body lesions, and there were no statistically significant differences among the three groups according to the RTOG CI, Nakamura’s CI, and HI [24]. Wu et al. reported the results from comparing 8–12 fields IMRT, 1-arc VMAT, and 2-arc VMAT plans for 10 cases of spine lesions. Paddick’s CI was only improved in 2-arc VMAT compared to 8–12 fields IMRT, and all other comparison indices (D_99_, D_95_, D_10_, D_5_, D_1_, and D_mean_) showed no statistically significant difference [3].

The basic concept of radiation therapy planning is the balancing of two opposing objectives: the target volume should be irradiated with as much of the prescribed dose as possible, and organs at risk should be spared from radiation as much as possible. Therefore, there are limitations to improve the dose coverage to the target volume while sparing the organs at risk. Among previously published studies, only a few reported that the indices for target volume and organs at risk could be improved simultaneously [18,32]. Other authors have reported the improvement of only one type of index [13-15,17].

In this study, all the treatment plans were designed to obtain the maximal target volume coverage satisfying the dose constraint of the spinal cord (maximum 10 Gy to 0.25 cc of spinal cord). Therefore, the irradiated dose to 0.25 cc of spinal cord was the same in all treatment plans. The present study was designed mainly to enable comparison of the indices for target volume, and thus, the comparison of the index for the spinal cord, which is the most important organ at risk in the treatment of the spinal lesions, was not necessary. In addition, to enhance the quality of the IMRT and VMAT plans, 11-field IMRT and 3-arc VMAT plans were carried out.

In this study, when the 5-mm MLC was replaced by the 2.5-mm MLC, TVC and GI improved significantly by 5.68% and 6.25%, respectively. With a smaller MLC leaf width, the improvement ratios of TVC were larger in IMRT than in VMAT (8.38% vs. 2.97%). Although the improvement was prominent in IMRT, the values of TVC were better in VMAT compared to IMRT. According to previous studies, 3D-CRT and DCAT are more sensitive to the MLC leaf width than IMRT [14,34,36]. We think that more sophisticated radiotherapy techniques cause higher dosimetric index values. Such techniques are less affected by the MLC leaf width. These trends were observed in our study as well.

The VMAT technique has wider low-dose regions than IMRT [38,39]. However, some authors have reported that VMAT does not always increase the regions of low-dose distribution compared to IMRT [2,40,41]. Lee et al. compared 7-field IMRT and 1-arc VMAT for spine metastases [2]. When prescribing 35 Gy, the V_10Gy_ for the remaining volume at risk was larger in IMRT than in VMAT (14.6% vs. 11%), which was statistically significant (*p* = 0.04). According to Wolff et al., in their study comparing VMAT and IMRT, V_30%_ was higher in 7-field IMRT than in 2-arc VMAT (3,414 cm^3^ vs. 3,340 cm^3^) [41]. However, in the case of 1-arc VMAT, V_30%_ was 3,438 cm^3^, which was higher than that in 7-field IMRT. In this study, V_30%_ was 460.80 cm^3^ in IMRT and 466.44 cm^3^ in VMAT, the difference of which is not statistically significant (*p* = 1.000). Lee et al. reported that the health tissue mean dose was larger in 18-field IMRT than in 7-field IMRT (29.37 Gy vs. 29.11 Gy) [42]. Therefore, the regions of low-dose distribution could differ on the basis of the number of fields and the type of parameter.

Several studies have reported that the micro-MLC and IMRT techniques are more effective when the target is small or its shape is complex [14,16-18,36]. According to the study of Kubo et al. on the effects of the MLC leaf width difference (1.7 mm, 3 mm, and 10 mm), when target shapes became more irregular, a smaller-width MLC leaf was more efficient [17]. Dhabaan et al. investigated the effect of the MLC leaf width difference (2.5 mm vs. 5 mm) and reported that when the target became small and had a complex shape, CI improved and the indices for organs at risk such as the conformity distance index, NTV50% (normal tissue volume ≥50% of prescription dose), NTV70%, and NTV90% also improved [18]. However, these researches were limited because the target complexities were not methodically classified.

In this study, the complexities of the target were classified into four types by using the WBB surgical staging system. The effects of the MLC leaf width were evaluated according to the type of target. Our study showed that a smaller MLC leaf width enhanced the target coverage. The improvement ratio of TVC in type 4, which was the most complex target shape, was 17.16%, and TVC was improved by 14.42-16.74% in type 4 compared to other types of targets. This means that as the target became more irregular, the dosimetric advantage of the smaller MLC increased. When a 2.5-mm MLC was used in VMAT, TVC was improved by only 6.34% in type 4, which was not statistically significant (*p* = 0.094). However, in IMRT, TVC was improved by 27.99% in Type 4, and the difference between type 4 and types 1–3 was 24.37-28.15%, which is statistically significant (*p* = 0.007). In addition, GI was significantly improved in IMRT (*p* = 0.047).

In conclusion, a smaller MLC leaf width provided improved target coverage in both IMRT and VMAT, and its improvement was larger in IMRT than in VMAT. In addition, a smaller MLC leaf width was more effective for a complex-shaped target.

## Competing interests

The authors declare that they have no competing interests.

## Authors’ contributions

SMC and SHS designed the experiment, and SMC performed the data collection. GWL performed the treatment planning and conducted all planning evaluations. SMC and SHS interpreted the data as well. SHS performed the statistical analysis. SMC drafted the manuscript. All authors have read and approved the final manuscript.
